# Case Report: A *de novo NLRP3* variant resulting in autoinflammatory disease in a Chinese newborn

**DOI:** 10.3389/fimmu.2023.1238551

**Published:** 2023-10-03

**Authors:** Mingyu Xie, Jingjing Wan, Xin Zheng, Xian Zou, Wanting Chen, Kanglin Zhang, Huiting Yuan, Zhenhong Zhang, Haisheng Zeng

**Affiliations:** ^1^ Department of Pediatric Rheumatology and Immunology, Dongguan Children’s Hospital, Dongguan, Guangdong, China; ^2^ Department of Pediatric Rheumatology and Immunology, Huizhou Central People’s Hospital, Huizhou, Guangdong, China

**Keywords:** autoinflammatory disease, NLRP3-AID, NLRP3, mutation, newborn

## Abstract

**Background:**

Cryopyrin-associated periodic syndromes (CAPS) have been considered autoinflammatory diseases resulting from *NLRP3* gene mutations. In recent years, these conditions have been redefined as *NLRP3*-associated autoinflammatory diseases (*NLRP3*-AID). Our previous study highlighted a case of a Chinese individual carrying the *de novo NLRP3* mutation.

**Results:**

A female child carrying a *de novo* variant (c.1718T>G, p. L573W) in the *NLRP3* gene was presented in this work. The patient manifested various symptoms, including recurrent fever, a rash resembling urticaria, arthritis, physical growth retardation, a notable prominence of the forehead, and a flat nose bridge. Additionally, inflammatory markers, like WBC count, PLT count, CRP, ESR, and IL-6 showed elevated levels. Additionally, we observed interstitial pulmonary disease in the patient, which is not frequently mentioned in previous studies. Notably, the proband did not present with any ocular, auditory, or neurological symptoms. After 12 weeks of subcutaneous canakinumab injection, there was a clear improvement in the patient’s clinical manifestations and inflammatory markers.

**Conclusion:**

Our study contributes to broadening the clinical spectrum of established pathogenic variants of *NLRP3* gene, which are related to NLRP3-AID.

## Background

Autoinflammatory diseases (AIDs) encompass various diseases resulting from innate immune system dysregulation, causing systemic effects (1). AIDs was first described by McDermott et al. in 1999 (2). As per the 2022 Classification of Monogenic Immune Disorders issued by the International Union of Immunological Societies (IUIS), there exists a total of 59 autoinflammatory disorder types resulting from mutations of 56 different genes, and this number is continuously expanding (3). Notably, among these conditions, *NLRP3-*AID was initially recognized in 2001 as the underlying factor leading to familial cold autoinflammatory syndrome (FCAS) (4). *NLRP3* gene is responsible for encoding cryopyrin protein, and its mutations commonly lead to three distinct periodic fever syndromes with varying severity: FCAS (OMIM 120100), Muckle-Wells syndrome (MWS) (OMIM 191900), as well as chronic infantile neurologic cutaneous articular syndrome (CINCA) (OMIM 607115) (5). To align with the 2018 Consensus Proposal for Taxonomy and Definition of Autoinflammatory Diseases, the term *NLRP3*-AID was recommended to encompass the spectrum of CAPS (6).

*NLRP3* variants can activate caspase-1, causing IL-1β (a pro-inflammatory cytokine) overexpression. Consequently, patients exhibit nonspecific clinical signs like urticaria-like rash, periodic fever, sensorineural deafness, arthritis, uveitis, and aseptic meningitis (5). Currently, over 170 *NLRP3* variants are identified, most of them are located within exon 3 that is responsible for encoding an oligomerization domain in cryopyrin protein (7). This research article presents a case study of an infant with *NLRP3*-AID who received treatment at our medical center. The clinical manifestations, medical progression, and subsequent monitoring of the affected individual are detailed herein.

## Case presentation

The proband, a second-born female child of Han Chinese parents, was delivered via cesarean section at 37 weeks gestation due to cloudy amniotic fluid. The birth weight and length were 2.2 kg and 48 cm separately.

**Figure 1:**
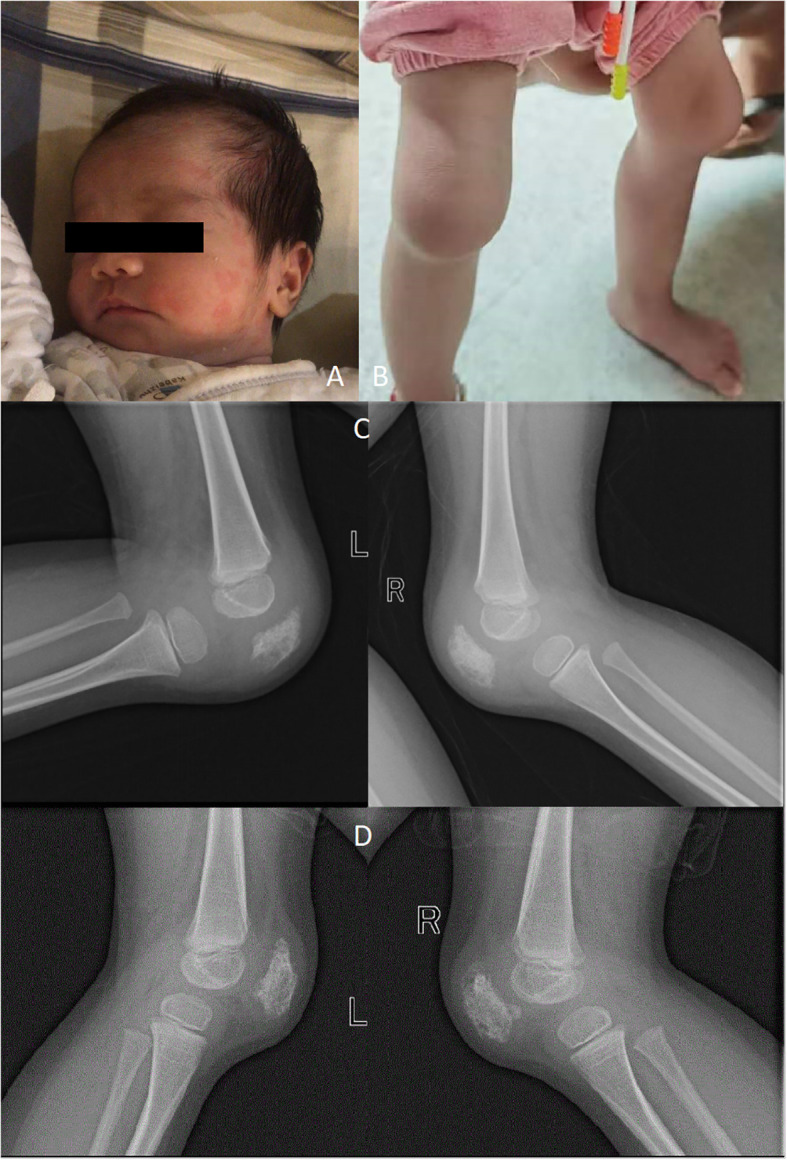
Rash **(A)**; Deformities of the knee **(B)**; X-ray showing destruction of patella **(C)**; X-ray of the knee were taken half a year after Canakinumab treatment **(D)**.

Shortly after birth, the proband experienced fever, accompanied by a red maculopapular rash that would fade upon pressure (**Figure 1A**). Neonatal pediatricians suspected a possible sepsis infection. The laboratory analyses revealed increased levels of acute-phase reactants during febrile episodes, with C-reactive protein (CRP) at 45.2 mg/L, procalcitonin (PCT) at 1.64 ng/mL, and interleukin-6 (IL-6) at 9.41 pg/mL. However, cultures of blood, urine, and stool yielded no growth. The proband’s body temperature would spontaneously return to normal, leading to a resolution of the rash. Without any obvious cause for the rash and fever, the parents opted to discharge the proband from the hospital. The proband continued to experience occasional rashes on a daily basis, with her weight and height measuring under the average for her age. When she was 1 years old, she also developed additional arthritis in her knees.

**Figure 2:**
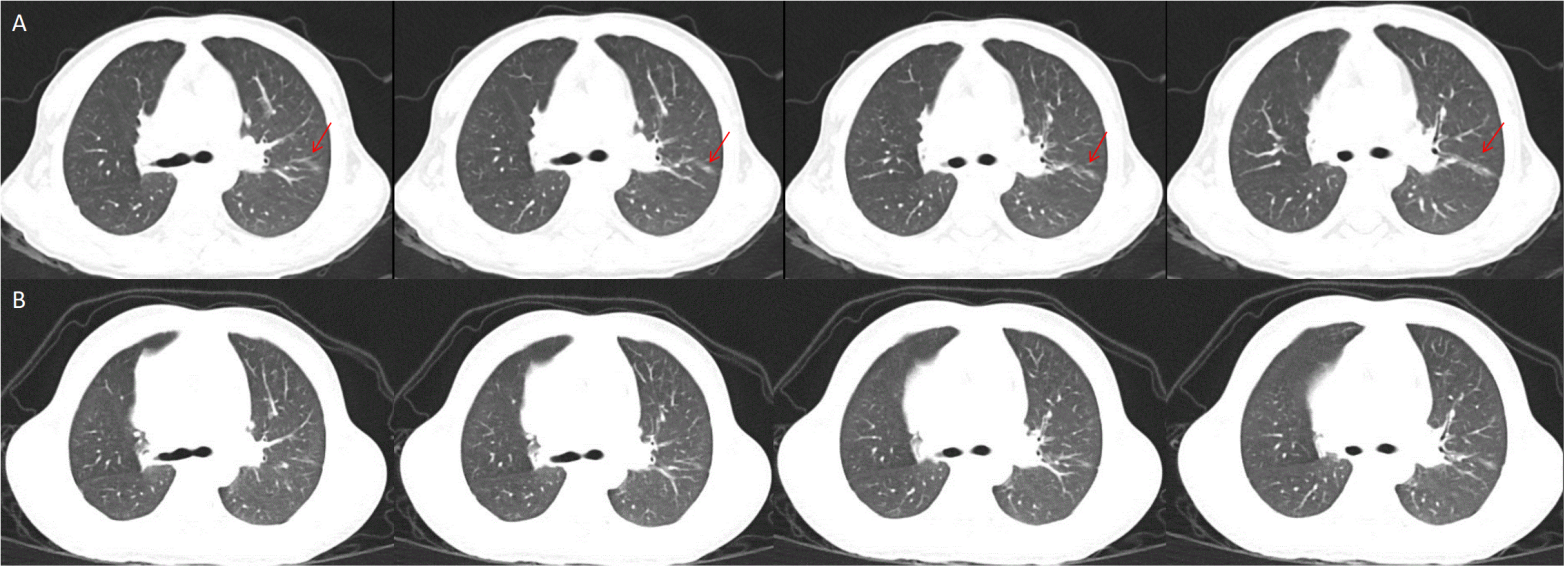
Interstitial lung disease in the left lung, the red snip point **(A)**, Pulmonary CT after eight months of Canakinumab treatment **(B)**.

When she was 2 years and 9 months old, she was admitted into Dongguan Children’s Hospital due to bilateral knee joint swelling and pain. Her height and weight were 82 cm and 8.7 kg (under the 3^rd^ percentile separately). In addition, the patient exhibited features such as a prominent forehead, flat nose bridge, rough skin, an unclosed anterior fontanelle, and enlarged knee joints (**Figure 1B**). The proband demonstrated normal intellectual and speech development. Laboratory tests revealed neutrophilic leukocytosis, thrombocytosis, increased acute phase reactants, and moderate microcytic hypochromic anemia. The white blood cell count (WBC) was 22.55 × 10^9^/L, with a neutrophil granulocyte count of 12.17 × 10^9^/L, hemoglobin (HGB) level of 98 g/L, and blood platelet (PLT) count of 466 × 10^9^/L. CRP levels were 66 mg/L, whereas the erythrocyte sedimentation rate (ESR) was 99 mm/h. Examination of peripheral blood cytokines demonstrated heightened levels of IL-6 at 32.02 pg/mL (range, 0–5.3 pg/mL), with IL-1β at 0.13 pg/mL (range, 0–3.4 pg/mL), IL-10 at 0.83 pg/mL (range, 0–4.91 pg/mL), and IL-12 at 1.62 pg/mL (range, 0–3.2 pg/mL). Rheumatoid factors (RF), antinuclear antibody profile (ANAs), human leukocyte antigen B27 (HLA-B27), as well as cyclic citrullinated peptide (CCP) were negative. Immunoglobulin levels showed IgA at 2.41 g/L, IgM at 17.7 g/L, and IgG at 2.33 g/L. Lymphocyte subset analysis indicated B and T cell activation. Ferritin levels were 43.34 ng/mL. The DR of the knees revealed patella bone destruction (**Figure 1C**). A chest CT scan showed interstitial lung disease in the left lung (**Figure 2A**), but she had no respiratory symptoms. Echocardiographic findings were normal. Nervous system MRI results were normal. The proband’s parents and older sister did not present similar clinical manifestations. The affected individual was given the diagnosis of juvenile idiopathic arthritis and subsequently received a treatment regimen of prednisone and methotrexate, as prescribed by a rheumatologist. However, her clinical symptoms exhibited only marginal improvement.

Whole exome sequencing (WES) analysis on this patient detected the *de novo* missense mutation (c.1718T>G, p. L573W) in exon 4 of *NLPR3* gene (NM_004895). Neither of the proband’s parents carried *NLRP3* gene mutations. Based on these findings, clinicians suggested that the proband was affected by an autoinflammatory disease caused by the NLRP3 genetic defect. Canakinumab (a monoclonal antibody against IL-1β), obtained from Hong Kong, was administered every 4 weeks to treat the autoinflammatory disease, guided by genetic testing. After one month of Canakinumab treatment, the fever, rash, and arthritis significantly improved. Inflammatory markers, including CRP, ESR, and IL-6, returned to normal levels after 12 weeks (**Table 1**). However, patellar bone destruction did not improve after half a year of Canakinumab treatment, and it merely showed a reduction in the soft tissue swelling around the knee joint (**Figure 1D**). Pulmonary CT showed that the interstitial changes of the left lung were slightly reduced after eight months of Canakinumab treatment (**Figure 2B**). The patient did not require the use of steroids or antirheumatic drugs at present. Her weight and height showed improvement, and she did not experience any adverse effects during the observation period.

**Table 1:** Changes in inflammatory markers before and after treatment with Canakinumab.

Time Blood examination indicator	WBC (×10^9/L)	HGB (g/L)	PLT (×10^9/L)	N (×10^9/L)	CRP (mg/L)	ESR (mm/h)	IL-6 (pg/ml)
Before treatment with Canakinumab	22.55	98	466	12.2	66	99	32.02
After 4 weeks of treatment with Canakinumab (The first dose)	16.88	111	321	8.47	21.7	65	17.24
After 8 weeks of treatment with Canakinumab (The second dose)	12.02	108	365	4.91	18.02	34	3.17
After 12 weeks of treatment with Canakinumab (The third dose)	10.8	109	301	3.87	0.5	12	2.19

## Materials and methods

### Study patient

This work was conducted at Dongguan Children’s Hospital, where demographic data, genetic results, and clinical presentations were documented using a Standardized Case Report Form.

### DNA extraction and WES

Peripheral blood was sampled in the affected individual together with her parents to extract genomic DNA with a Magen SolPure Blood DNA kit, in line with specific guidelines. Next-generation sequencing was employed to sequence the proband’s DNA, while first-generation sequencing was used to confirm the parents’ DNA based on the proband’s genetic findings.

### Bioinformatic analysis of the variants

Sequence variant annotation was carried out with various literature and population databases, which included 1000 Genomes (http://www.1000genomes.org/), gnomAD (http://gnomad.broadinstitute.org/), dbSNP (http://www.ncbi.nlm.nih.gov/), ClinVar (https://www.ncbi.nlm.nih.gov/clinvar/), OMIM (https://www.omim.org/) and HGMD (http://www.hgmd.cf.ac.uk/ac/index.php). Protein structure analysis, prediction of functional and conserved domains, along with multiple sequence alignment were completed using online software (https://www.uniprot.org/). Variant interpretation followed the guidelines provided by the American College of Medical Genetics (ACMG) (8). Additionally, web tools such as PolyPhen-2, MutationTaster, MutationAssessor, and SIFT, were utilized to predict potential pathogenicity of the variants.

## Results

### Genetic analysis

**Figure 3:**
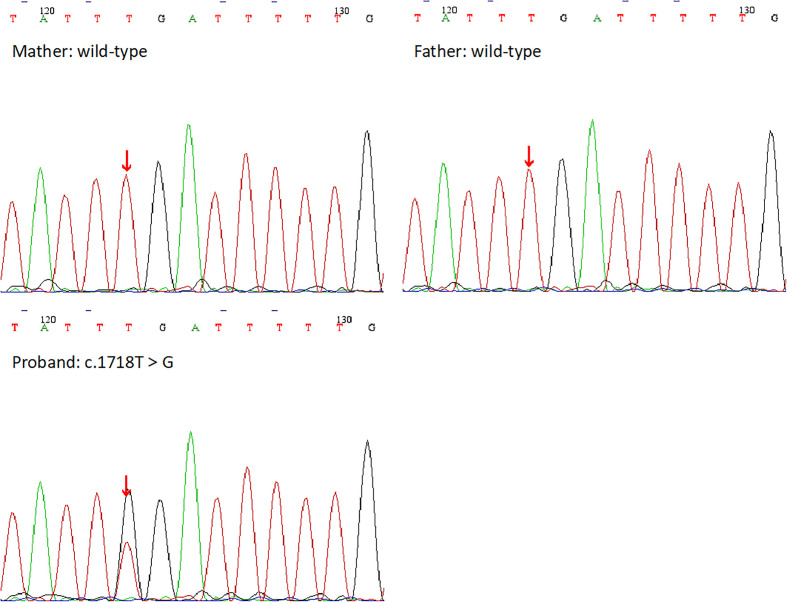
Schematic representation of the NLRP3 protein. Most previously reported mutations are located in NACHT domain, and the novel mutation is in a non-functional domain.

**Figure 4:**
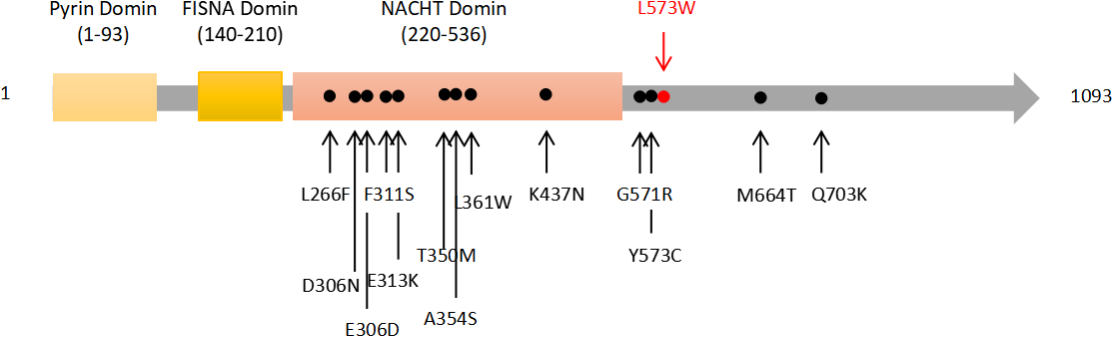
The DNA sequencing results of the proband and her family. Her parents showed the wild-type at the base of c.1718T, but she showed a heterozygous mutation c.1718T>G.

The WES results demonstrated the missense mutation, c.1718T>G (p.L573W), within exon 4 in *NLPR3* gene (NM_004895) of this patient. The particular mutation, c.1718T>G (p.L573W), was absent within gnomAD, ExAC, as well as 1000 Genomes Project databases. Furthermore, *NLRP3* gene mutation was not detected in her parents (**Figure 3**). The missense mutation, c.1718T>G (p.L573W), was located adjacent to NACHT-NTPase domain in NLRP3 protein (**Figure 4**). Furthermore, our analysis revealed that the amino acid residue affected by the mutation, L573, was highly conserved across different species. Prediction tools indicated that the variant *NLRP3* was classified as “disease causing” by MutationTaster, “affecting protein function” by SIFT, and “probably damaging”, and the PolyPhen-2 score was 0.999 (sensitivity and specificity of 0.00 and 1.00 separately). Additionally, MutationAssessor categorized the mutation as having a “high” impact on protein function.

## Discussion and conclusions

As of now, around 124 pathogenic or possibly pathogenic *NLRP3* gene variants have been recognized (www.ncbi.nlm.nih.gov/clinvar/). Most of these variants are situated within exon 3, responsible for encoding “NLR-binding” domain in cryopyrin protein. These variants have been linked to three primary clinical syndromes: FCAS, MWS, and CINCA. FCAS shows the features of rash and fever without eye or hearing involvement. In contrast, MWS demonstrates the features of an urticaria-like rash unrelated to cold exposure, prominent skeletal manifestations, and severe hearing loss. CINCA is diagnosed when a newborn presents with rash, chronic meningitis, arthropathy, fever, and inflammation (9). The most common clinical signs observed in patients with NLRP3-associated autoinflammatory diseases are fever, rash, and elevated levels of ESR, CRP, and IL-6 (**Table 2**). Previous study indicated that half of children’s patients with CAPS exhibited neurologic manifestations with varying degrees of severity, including headache, papilledema, sensorineural deafness, epilepsy, hypophrenia, hydrocephalus or aseptic meningitis (14). Headache was the most common neurological symptom (15). However, in our study, we identified a *de novo NLRP3* variant, c.1718T>G (p.L573W), in a patient who did not exhibit any neurological symptoms other than physical growth retardation. Of course, we couldn’t not exclude the possibility that the proband was unable to accurately describe her symptoms because of her younger age. The characteristic of lower frequency neurological symptom in younger patients has also been reflected in previous reports (15). We speculated that neurological symptoms might be associated with the time of diagnosis and clinical intervention. Patients with higher frequency of neurological symptoms were older age at diagnosis (9, 15).

**Table 2:** Clinical manifestations of previously reported patients with various NLRP3 mutations and the novel NLRP3-AID.

No.	location	Clinical signs								laboratory examination				imageological examination		Reference
		Fever	rash	arthritis/joint deformity/frontal bossing	Growth retardation	Neurological symptoms	Hearing loss	Ocular symptoms	Musculoskeletal manifestations	Leukocytosis	Anemia	Thrombocytosis	Increased CRP/SR/IL-6	Interstitial lung disease	difuse brain atrophy with cortical calcifcations	
1	c.796C>T, p.L266F	√	√			√				√		√	√			(9)
2	c.913G>A, p.D305N	√	√		√	√	√	√	√	√	√	√	√			(9)
3	c.918G>T, p.E306D	√	√	√		√	√		√	√	√	√	√			(9)
4	c.932T>C, p.F311S	√	√			√				√	√	√	√			(9)
5	c.G937A p.E313K	√	√	√			√	√	√							(10)
6	c.1049C>T, p.T350M	√	√		√					√			√			(9)
7	c.1060G>A,p.A354S	√	√	√	√					√	√	√	√			(11)
8	c.1082T>G, p.L361W	√	√			√		√	√	√	√	√	√			(9)
9	c.1311G>T, p.K437N	√	√	√	√	√			√	√		√	√			(9)
10	c.1711G>A, p.G571R	√	√				√			√		√	√			(9)
11	c.1715A>G, p.Y572C		√	√		√	√		√	√	√	√	√			(9)
12	c.1718T>G, p. L573W	√	√	√	√					√	√	√	√	√		Our case
13	c.1872C>T, p.S624R	√	√	√	√	√	√	√	√	√	√	√	√		√	(12)
14	c.1991T>C, p.M664T	√	√	√	√	√	√	√	√	√	√	√	√		√	(9)
15	c. 2107C>A, p.Q703K	√	√	√		√	√		√				√			(13)

Although an accurate genotype-phenotype correlation has not yet been established, *de novo* mutations can be often related to serous CINCA phenotype (16). In our study, the proband who did not exhibit the typical CINCA phenotype, such as hearing loss, optic neuritis or severe central nervous system manifestations. Previous reports have described patients with *de novo NLRP3* variants, such as c.932T>C (p.F311S), c.796C>T (p.L266F), c.1311G>T (p.K437N), c.1711G>A (p.G571R), and c.1049C>T (p.T350M), who showed milder phenotypes resembling FCAS and MWS (9). This difference in phenotype may be attributed to earlier diagnosis or intervention with appropriate treatment. Our findings indicate that the clinical phenotype of NLRP3-associated autoinflammatory diseases is not solely determined by the specific domain or location of the mutated amino acid in NLRP3. Although most documented NLRP3 mutations are situated in the NACHT domain, they can give rise to various clinical phenotypes, encompassing FCAS, MWS, or CINCA. Additionally, it is worth noting that the same variant can lead to different clinical phenotypes (9). This highlights the importance of a comprehensive evaluation by clinicians when assessing patients with *NLRP3*-AID.

*NLRP3*-AID is often misdiagnosed as juvenile idiopathic arthritis due to shared symptoms such as fever, rash, and joint inflammation. However, most patients with NLRP3-AID do not exhibit increased levels of ferritin which can help clinicians differentiate between the two conditions (9). *NLRP3*-AID may sometimes be misdiagnosed as other febrile cycle syndromes, such as Familial Mediterranean fever (FMF) and periodic fever, aphthous stomatitis, pharyngitis, and adenitis (PFAPA) syndrome. FMF represents the monogenic autoinflammatory disorder resulting from *MEFV* variants. Initially considered an autosomal recessive disorder, recent studies have indicated about 25% dominant inheritance (17). FMF shares a similar pathogenesis with *NLRP3*-AID, as both conditions involve pyrin deficiency causing caspase-1 activation together with proinflammatory factor generation like IL-1β (18). However, FMF shows the typical clinical features of fever, serositis, erysipelas-like erythema and arthritis (19). For our case, DNA sequencing results ruled out the presence of any MEFV mutation in the proband. On the other hand, PFAPA is a polygenic disease strongly associated with genetic variants close to *IL12A, IL10, STAT4*, as well as *CCR1*–*3* genes (20). The clinical features of PFAPA generally include periodic high fever (ranging from 39°C to 40°C), pharyngitis, cervical adenitis, aphthous ulcers, and mild abdominal/muscle pain (21). Clearly, these clinical manifestations do not match the symptoms observed in our case. Furthermore, we observed a rare presentation in our case study. A chest CT scan revealed mild interstitial lung disease, which has not been previously reported in similar case studies.

IL-1 Receptor antagonists, such as canakinumab, anakinra, and rilonacept, are safe and effective for long-term treatment (5). In this instance, our case received canakinumab therapy at 2 mg/kg every 28 days. Canakinumab accounts for the monoclonal antibody targeting IL-1β by specifically binding to soluble IL-1β (5). Notably, four weeks following the administration of canakinumab injections, the patient exhibited substantial improvement in their clinical symptoms. By week 12, the inflammatory markers had returned to near-normal levels. The patient’s height and weight also improved with proper control of these inflammatory markers. Nonetheless, an extended observation period should be necessary for assessing the long-time effect and potential adverse effects of the treatment.

The *NLRP3* variant c.1718T>G (p.L573W) identified in our study may contribute to the development of *NLRP3*-AID. This novel variant adds to the existing spectrum of known *NLRP3* mutations in human populations. However, further mechanistic studies are crucial for developing novel treatments that target *NLRP3*-AID.

## Data availability statement

The original contributions presented in the study are included in the article/supplementary files, further inquiries can be directed to the corresponding author/s.

## Ethics statement

The studies involving humans were approved by Ethics Committee of Dongguan Children’s Hospital. The studies were conducted in accordance with the local legislation and institutional requirements. The human samples used in this study were acquired from another research group. Written informed consent for participation was not required from the participants or the participants’ legal guardians/next of kin in accordance with the national legislation and institutional requirements. Written informed consent was obtained from the individual(s), and minor(s)’ legal guardian/next of kin, for the publication of any potentially identifiable images or data included in this article.

## Author contributions

MX and JW were in charge of data collection and manuscript drafting. XZ, XAZ, WC, KZ, ZZ, and HZ were responsible for study conception, design, manuscript writing, checking and revision. All authors contributed to the article and approved the submitted version.

## References

[1] Krainer J Siebenhandl S Weinhäusel A. Systemic autoinflammatory diseases. J Autoimmun (2020) 109:102421. doi: 10.1016/j.jaut.2020.102421 32019685 PMC7610735

[2] McDermott MF Aksentijevich I Galon J McDermott EM Ogunkolade BW Centola M. Germline mutations in the extracellular domains of the 55 kDa TNF receptor, TNFR1, define a family of dominantly inherited autoinflammatory syndromes. Cell (1999) 97(1):133–44. doi: 10.1016/s0092-8674(00)80721-7 10199409

[3] Tangye SG Al-Herz W Bousfiha A Cunningham-Rundles C Franco JL Holland SM. Human inborn errors of immunity: 2022 update on the classification from the international union of immunological societies expert committee. J Clin Immunol (2022) 42(7):1473–507. doi: 10.1007/s10875-022-01289-3 PMC9244088 35748970

[4] Hoffman HM Mueller JL Broide DH Wanderer AA Kolodner RD. Mutation of a new gene encoding a putative pyrin-like protein causes familial cold autoinflammatory syndrome and Muckle-Wells syndrome. Nat Genet (2001) 29(3):301–5. doi: 10.1038/ng756 PMC4322000 11687797

[5] Welzel T Kuemmerle-Deschner JB. Diagnosis and management of the cryopyrin-associated periodic syndromes (CAPS): what do we know today? J Clin Med (2021) 10(1):128. doi: 10.3390/jcm10010128 33401496 PMC7794776

[6] Ben-Chetrit E Gattorno M Gul A Kastner DL Lachmann HJ Touitou I. Consensus proposal for taxonomy and definition of the autoinflammatory diseases (AIDs): a Delphi study. Ann Rheum Dis (2018) 7(11):1558–65. doi: 10.1136/annrheumdis-2017-212515 30100561

[7] Milhavet F Cuisset L Hoffman HM Slim R El-Shanti H Aksentijevich I. The infevers autoinflammatory mutation online registry: update with new genes and functions. Hum Mutat (2008) 29(6):803–8. doi: 10.1002/humu.20720 18409191

[8] Richards S Aziz N Bale S Bick D Das S Gastier-Foster F. Standards and guidelines for the interpretation of sequence variants: a joint consensus recommendation of the American College of Medical Genetics and Genomics and the Association for Molecular Pathology. Genet Med (2015) 17:405–24. doi: 10.1038/gim.2015.30 PMC4544753 25741868

[9] Zhou Y Wang W Zhong L Wang L Ma M Tang X. Clinical and genetic spectrum of 14 cases of NLRP3-associated autoinflammatory disease (NLRP3-AID) in China and a review of the literature. Orphanet J Rare Dis (2022) 7(1):214. doi: 10.1186/s13023-022-02364-z PMC9169254 35668534

[10] Chen P He L Pang X Wang X Yang T Wu H. NLRP3 is expressed in the spiral ganglion neurons and associated with both syndromic and nonsyndromic sensorineural deafness. Neural Plast (2016) 2016:3018132. doi: 10.1155/2016/3018132 27965898 PMC5124661

[11] Vahedi M Parvaneh N Vahedi S Shahrooei M Ziaee V. Identification of a new variant in NLRP3 gene by whole exome sequencing in a patient with cryopyrin-associated periodic syndrome. Case Rep Immunol (2021) 2021:2023119. doi: 10.1155/2021/2023119 PMC8384535 34447596

[12] Ducharme-Bénard S Roberge G Chapdelaine H. A unique presentation of NLRP3-associated autoinflammatory disease: case report. BMC Rheumatol (2022) 6(1):91. doi: 10.1186/s41927-022-00321-8 36510304 PMC9743682

[13] Naselli A Penco F Cantarini L Insalaco A Alessio M Tommasini A. Clinical characteristics of patients carrying the Q703K variant of the NLRP3 gene: A 10-year multicentric national study. J Rheumatol (2016) 43(6):1093–100. doi: 10.3899/jrheum.150962 27036377

[14] Kilic H Sahin S Duman C Adrovic A Barut K Turanli ET. Spectrum of the neurologic manifestations in childhood-onset cryopyrin-associated periodic syndrome. Eur J Paediatr Neurol (2019) 23(3):466–72. doi: 10.1016/j.ejpn.2019.03.006 30967326

[15] Parker T Keddie S Kidd D Lane T Maviki M Hawkins PN. Neurology of the cryopyrin-associated periodic fever syndrome. Eur J Neurol (2016) 23(7):1145–51. doi: 10.1111/ene.12965 26931528

[16] Levy R Gérard L Kuemmerle-Deschner J Lachmann HJ Koné-Paut I Cantarini L. Phenotypic and genotypic characteristics of cryopyrin-associated periodic syndrome: a series of 136 patients from the Eurofever Registry. Ann Rheum Dis (2015) 74(11):2043–9. doi: 10.1136/annrheumdis-2013-204991 25038238

[17] Ozdogan H Ugurlu S. Familial mediterranean fever. Presse Med (2019) 48(1 Pt 2):e61–76. doi: 10.1016/j.lpm.2018.08.014 30686512

[18] Schnappauf O Chae JJ Kastner DL Aksentijevich I. The pyrinInflammasome in health and disease. Front Immunol (2019) 7:1745(10). doi: 10.3389/fimmu.2019.01745 PMC6698799 31456795

[19] Rigante D Lopalco G Tarantino G Compagnone A Fastiggi M Cantarini L. Non-canonical manifestations of familial Mediterranean fever: a changing paradigm. Clin Rheumatol (2015) 34(9):1503–11. doi: 10.1007/s10067-015-2916-z 25761640

[20] Manthiram K Preite S Dedeoglu F Demir S Ozen S Edwards KM. Common genetic susceptibility loci link PFAPA syndrome, Behçet’s disease, and recurrent aphthous stomatitis. Proc Natl Acad Sci U S A (2020) 117(25):14405–11. doi: 10.1073/pnas.2002051117 PMC7322016 32518111

[21] Wang A Manthiram K Dedeoglu F Licameli GR. Periodic fever, aphthous stomatitis, pharyngitis, and adenitis (PFAPA) syndrome: A review. World J Otorhinolaryngol Head Neck Surg (2021) 7(3):166–73. doi: 10.1016/j.wjorl.2021.05.004 PMC8356195 34430824

